# Pathogens associated with persistent diarrhoea in children in low and middle income countries: systematic review

**DOI:** 10.1186/1471-2334-9-88

**Published:** 2009-06-10

**Authors:** Katharine Abba, Rebecca Sinfield, C Anthony Hart, Paul Garner

**Affiliations:** 1 International Health, Liverpool School of Tropical Medicine, Pembroke Place, Liverpool, L3 5QA, UK; 2 Arrowe Park Hospital, Arrowe Park Road, Upton, Wirral, Merseyside, CH49 5PE, UK

## Abstract

**Background:**

Persistent diarrhoea in children is a common problem in low and middle income countries. To help target appropriate treatment for specific pathogens in the absence of diagnostic tests, we systematically reviewed pathogens most commonly associated with persistent diarrhoea in children.

**Methods:**

We sought all descriptive studies of pathogens in the stool of children with diarrhoea of over 14 days duration in low and middle income countries with a comprehensive search of the MEDLINE, EMBASE, LILACS and WEB OF SCIENCE databases. We described the study designs and populations, assessed the quality of the laboratory tests, and extracted and summarised data on pathogens. For *Escherichia coli*, we calculated high and low prevalence estimates of all enteropathic types combined. Results across studies were compared for geographical patterns.

**Results:**

Nineteen studies were included. Some used episodes of diarrhoea as the unit of analysis, others used children. The quality of reporting of laboratory procedures varied, and pathogens (particularly *E. coli *types) were classified in different ways. As there were no apparent regional differences in pathogen prevalence, we aggregated data between studies to give a guide to overall prevalence. Enteropathic *E. coli *types were commonly found in children with persistent diarrhoea (up to 63%). Various other organisms, including viruses, bacteria and parasites, were detected but across all studies their prevalence was under 10%. However, these pathogens were also found in similar frequencies in children without diarrhoea.

**Conclusion:**

A number of pathogens are commonly associated with persistent diarrhoea in children, but in children without diarrhoea the pathogens are found with similar frequencies. New research with carefully selected controls and standardised laboratory investigations across countries will help map causes and help explore effective options for presumptive treatment.

## Background

### The problem

In 2002, diarrhoea caused an estimated 13.2% of child deaths worldwide[1], most of them in children under the age of five in low and middle income countries[2]. In this group of children, around 3% to 19% of acute diarrhoea episodes become persistent[3], and some experts estimate that up to 50% of diarrhoea deaths may be due to persistent diarrhoea[1]. As the number of deaths from acute diarrhoea reduces following the widespread use of oral rehydration therapy, the contribution of persistent diarrhoea to overall diarrhoea mortality is increasing. In addition, persistent diarrhoea may adversely affect nutritional status[4] and is often associated with malnutrition.

Children living in poor areas with poor hygiene and sanitation conditions and children with poor nutritional status are most at risk of developing persistent diarrhoea[3]. As poor nutrition is both a risk factor and a consequence of persistent diarrhoea, the two are very commonly associated. Children with HIV/AIDS are at particular risk; at initial presentation to hospital with HIV/AIDS, around 36–50% [5-7] of children have persistent diarrhoea. Dysentery and more severe diarrhoeal illnesses are more likely to become persistent than milder episodes[3]. Previous antibiotic use and irrational use of antibiotics are also considered to be risks factors for persistent diarrhoea[3].

### Definition and causes

The World Health Organization (WHO) defines diarrhoea as the passing of three or more loose stools (which take the shape of the container) within a 24 hour period. A new episode of diarrhoea can occur after two full days without diarrhoea. Episodes of diarrhoea lasting for less than 14 days are defined as acute, episodes lasting for more than 14 days are defined as persistent.

The causes of persistent diarrhoea in populations are poorly understood, and in individuals often unknown. Some pathogens, such as *Cryptosporidium*, *Giardia lamblia *and enteroaggregative *Escherichia coli *(EAggEC) are thought to be associated with persistent diarrhoea in some locations[3]. Children with persistent diarrhoea and HIV infection may have different patterns of enteric pathogens than those without HIV[8]. Pathogens detected in persistent diarrhoea are often not the same as those detected in the original acute episode, suggesting that secondary infections may be important[9]. In addition, children may be infected with more than one enteric pathogen, making it difficult to identify which, if any, is causing the illness, or may have no detectable enteric pathogens. Persistent diarrhoea may also be associated with bacterial overgrowth in the small bowel[10], and with poor nutritional status[11]. In addition, the diarrhoea may be caused by multiple factors including micronutrient deficiencies, milk or food intolerances, or diseases of the bowel, as well as prior antibiotic therapy[12].

### When and how to treat

The current recommendations of the Integrated Management of Childhood Illness programme[13] for treating persistent diarrhoea is that children with bloody diarrhoea are treated with antibiotics for *Shigella*, or for *Entamoeba histolytica *where the organism is detected in the stool; it is recommended that children with watery diarrhoea are not treated with antimicrobials; except where *Giardia lamblia *is found.

Even when an enteric pathogen is detected in children with persistent diarrhoea, it is not always clear that this is the cause of the illness. In addition, health workers in low and middle income countries often have limited access to diagnostic facilities to analyse stool samples of children with diarrhoea. In these situations, treatment needs to be syndromic, based on symptoms and the most likely cause of the symptoms; and may include replacement fluid and electrolytes, nutritional rehabilitation and sometimes drug treatment[12].

The use of antimicrobials presumptively needs to be approached with caution, as this can lead to drug resistance, and also to potential adverse reactions with some micro-organisms: entero-haemorrhagic *E. coli *(EHEC) may release toxins more readily when a person is treated with certain types of antibiotic, potentially causing severe illness[14].

Given the limited knowledge about the causes of persistent diarrhoea, we carried out a systematic review of studies that have attempted to identify aetiological agents in young children with this condition in low and middle income countries. We hoped in particular to identify whether any particular organisms were commonly associated with this condition, and if there were any geographic patterns. Either could help guide treatment of this condition in the absence of diagnostic facilities. The objective of this review was to summarise studies of pathogens reported in children with persistent diarrhoea and, where available, in appropriate comparison groups without diarrhoea; and to explore whether there are any patterns in the findings by region.

## Methods

### Inclusion criteria

We searched for published descriptive studies that analysed routinely collected data, data collected as part of a surveillance project or point survey data, and which provided data on pathogens detected in the stool of at least 30 children under the age of six years, living in a low or middle income country and having persistent diarrhoea (over 14 days duration). In reports where the age range of participants was not specifically stated, but it was apparent that all or almost all participants were below the age of six, these studies were included. Participants could be recruited from any setting including the general community, primary health care services, and hospital outpatient and inpatient services. Studies could also include an appropriate comparison group of children without diarrhoea.

Studies also including older children or adults, or diarrhoea lasting for less than 14 days, were included provided that data from observations specific to children under the age of six with persistent diarrhoea could be extracted. Studies presenting data on only one species of pathogen were excluded.

### Search strategy

The search strategy was developed in collaboration with an information retrieval specialist (see below). The strategy was amended where necessary to search the other databases listed. No language restrictions were applied. The reference lists of included studies were also scrutinised for additional relevant studies, and an informal search was undertaken of 'Google Scholar' using the search terms 'pathogens persistent diarrhoea'. The final formal search was undertaken May 20, 2008.

#### Search strategy

Databases searched:

MEDLINE (1966 to date) via the OVID interface

EMBASE (1980 to date) via the OVID interface

LILACS database – Latin American and Caribbean Health Sciences Literature (1982 to present) – via Virtual Health Library interface

WEB OF SCIENCE (Science Citation Index Expanded – 1945 to present)

The following search strategy was used in MEDLINE:

1. persistent (diarrhoea OR diarrhea) ti, ab.

2. chronic (diarrhoea OR diarrhea) ti, ab.

3. watery (diarrhea OR diarrhea) ti, ab

4. (diarrheal disease*) OR (diarrhoeal disease*) ti, ab

6. 1 OR 2 OR 3 OR 4) NOT cancer NOT (inflammatory bowel disease*) NOT (ulcerative colitis)

7. diarrhea, Infantile/classification OR diarrhea, infantile/etiology OR diarrhea, infantile/microbiology OR diarrhea, infantile/parasitology OR diarrhea, infantile/pathology [MeSH]

8. 6 OR 7

9. child* OR infant* OR pediatr* ti, ab

10. 8 AND 9

11. case-control studies [MeSH] OR epidemiological studies [MeSH]

12. descript* OR surve* OR monitor* ti, ab

13. 11 OR 12

14. 10 AND 13, Limits:humans

### Study selection

Two authors independently inspected titles and abstracts identified by the literature search in order to identify potentially relevant publications. All potentially relevant publications identified by at least one reviewer were obtained in full text format, with the exception, due to time constraints, of reports not available within the United Kingdom. One author then applied the inclusion criteria to select which studies to include in the review, consulting with another in cases of uncertainty. All publications were scrutinised for duplication of study results.

#### Data extraction

For each study report, two authors worked together to extract information describing the characteristics of the study and assess its quality, using a proforma as a guide. The data items extracted are shown below.

#### Data extracted from included studies

Geographic location of the study, including relevant socio-economic or environmental situation, and WHO region

Start and end dates of the study, and duration

Age range of the participants, and any additional inclusion criteria or local factors such as HIV infection or malnutrition. Where the participants were recruited from, for example, outpatient clinic, hospital ward, community, and how they were sampled

Whether or not the study also had a comparison group without diarrhoea, how this group were selected and whether and how they were matched to the diarrhoea group

Sample sizes of the persistent diarrhoea group and comparison group with no diarrhoea

Unit of analysis; whether data was presented by children or episodes

Types of enteric pathogen tested for in each group (persistent diarrhoea and comparison group without diarrhoea)*

Number of children (or episodes) tested for each pathogen*

Number or percentage of children, episodes or samples where each pathogen was detected*

Number or percentage of children, episodes or samples with any detected enteric pathogen*

*One author extracted this data.

We extracted data on selected pathogens only, which we identified as important potential causes of diarrhoea in terms of both prevalence and potential to cause disease. The list of pathogens for which we intended to extracted data, where available, is shown in Table 1. We extracted, where available, data on 'any pathogen' only where studies tested for a reasonably comprehensive range of pathogens. Where any organisms presented in a study paper were excluded from our analysis, we did not use data presented on 'any pathogen' from that study.

**Table 1:** List of pathogens for which data was extracted

Viruses	Bacteria	Parasites
Rotavirus	Campylobacter	Giardia lamblia
Enteric adenovirus	Shigella	Crytosporidium
Astrovirus	Salmonella	Entamoeba histolytica
Norovirus	Vibrio cholera	Microsporidia
	Enteropathic E coli (excluding EHEC)	Isospora
	Entero haemorrhagic E. coli (EHEC)	Cyclospora

For *E. coli*, we combined all enteropathic types (any which have the capacity to cause enteric illnesses), with the exception of the entero-haemorrhagic type (EHEC), into one category, because different studies classified *E. coli *very diversely, making it impossible to compare types across studies. *E. coli *of an unspecified type, or classed as 'untypable' was excluded from the analysis because it was not possible to assess whether it was enteropathic. *E. coli *described by the authors as diff. *E. coli*, EAEC-D, EAEC (diffuse) or *E. coli *showing diffuse Hep-2 adherence were also excluded due to uncertainty about whether or not they are pathogenic. EHEC was kept separate where possible due to its different behaviour when exposed to antibiotic drugs. For each study a note was made of the *E. coli *types tested for, as described in the study.

Where it was not clear in a report whether some individuals may have tested positive for more than one type of *E. coli*, we calculated high and low estimates of overall enteropathic *E. coli *frequency, using the largest *E. coli *category as a conservative estimate and all *E. coli *categories added together as a high estimate, adjusted where appropriate using any additional information presented in the report (for example, number of participants with any pathogens detected, number with more than one pathogen detected).

We planned to also extract data for any subgroups presented in the reports (for example by age or HIV status), but no reports presented data for subgroups.

One author also extracted data to describe the quality of reporting of the laboratory procedures used for the study, and whether any of the pathogens were tested for antimicrobial sensitivity. The data items extracted are shown in Table 2.

**Table 2:** Assessment of laboratory procedures

Laboratory processes:	**Samples**: Were there any methods or timings described for collecting, storing and transporting stool samples?
	**Parasite detection**: Were any methods described or referenced for the detection of parasites, other than the word 'microscopy'? (e.g. stain, magnification)?
	**Bacterial Culture**: Were any methods described or referenced for the culture of bacteria, other than the word 'culture'? (e.g. media, temperature)
	**Bacteria identification**: Were any methods described or referenced for the identification of cultured bacteria?
	**Virus detection**: Were any methods described for the identification of viruses? (the name of the test was sufficient)
Quality control	Were methods described for testing and controlling the quality of the laboratory tests used in the study?

Antimicrobial susceptibility testing	Which of the pathogens detected in the study, if any, were tested for antimicrobial susceptibility?

### Data analysis

For each relevant reported pathogen in each study, we extracted or calculated the number and percentage of children, in both the persistent diarrhoea group and comparison group without diarrhoea, with the pathogen detected in the stool, based on the number of cases or individuals tested and the number found positive. We also calculated the standard deviation and the 95% confidence intervals around the percentage.

Studies were grouped by the WHO Region of the study population. For each region, and for all the studies together, we noted or calculated the following parameters for each pathogen and for any pathogen in combined groups with persistent diarrhoea:

Number of studies where the pathogen was tested for and results presented

Total number of participants or episodes where the pathogen was tested for and results presented

The maximum and minimum percentage across studies of children or episodes where the pathogen was detected

The median percentage across studies of children or episodes where the pathogen was detected

Mean percentage across studies (weighted by study sample size) of children or episodes where each pathogen was detected

For the comparison groups without diarrhoea we calculated similar summary statistics. However, in order to make the mean percentage across the studies comparable with that for the persistent diarrhoea group, we weighted the contribution of each study according to the size of the group with persistent diarrhoea, rather than the group without diarrhoea. We did not calculate confidence intervals for statistics derived from combined studies.

We do not present any data based on a combined number of diarrhoea cases or comparisons of less than 30.

## Results

### Selection of studies

The initial search returned 1,853 references, from which we selected 102 for full text retrieval; of these we were able to retrieve 98; four were not available within the United Kingdom and were therefore not included due to lack of time and resources. From these 98 reports we selected 14 studies for inclusion in the review. Table 3 lists the reasons for exclusions of reports not included, and the number in each category. We found reports of a further three relevant studies through the reference lists of included studies, another two through a different search undertaken for another, related review, and three through an informal search of 'Google Scholar'. We included a total of 20 reports describing 19 studies[4,15-33]. Two reports were from the same surveillance project but presented data on different pathogens[15,16], and also included children of a slightly different age range.

**Table 3:** Reasons for exclusion of reports identified in the initial screening process as potentially relevant

Reason for exclusion	Number of Reports
Diarrhoea status of participants	

Included children with acute diarrhoea only	9
Wrong definition of persistent diarrhoea	2
Asymptomatic children only	1
Included all children in a community, whether or not they had diarrhoea	2
No separate analysis for cases of acute and persistent diarrhoea	41

Other participant characteristics	

Participants were of the wrong age range	8
Participants resided in a high income country	3

Study design factors	

Less than 30 participants	5
Faecal samples taken in the acute stage (before day 14) of the persistent episode	5
Tested for one organism only	4
Sampled only children who had been in close contact with *Shigella *dysentery	1
Reviewed hospital records only for children found positive for enteric pathogens	1

Not relevant	

No data on frequency of pathogens presented	5

### Characteristics of included studies

Details of the characteristics of the individual studies included in the review are presented in Tables 4 and 5 (link). Table 4 shows the characteristics of all the included studies, in terms of the groups with persistent diarrhoea, and Table 5 shows only the eight studies with a comparison group without diarrhoea[4,17-20,23,27,28,30], in terms of the comparison group. One paper[32] did present findings for a comparison group, but this group was excluded from the analysis because it only included 15 individuals. Two papers based on the same data set are described separately as they present data on a slightly different age group[15,16]. Within each table, the studies are arranged by WHO region where they were carried out. Each study is named according to the country where it was conducted and the year of publication. A summary of the information presented in Tables 4 and 5 is presented below.

**Table 4:** Characteristics of included studies: groups with persistent diarrhoea

Study report	Location	Start Duration	Participants	Source of cases	Sampling method	Number of cases	Unit of Analysis
Bangladesh 1992a [16]	Matlab (rural)	1988 1 yr	< 5 yrs	Active household surveillance	All cases identified	Varied by pathogen (68 – 184)	Episodes
Bangladesh 1992b [17]	Mirzapur (rural)	1987 2 yrs	< 6 yrs	Active household surveillance	All cases identified	Varied by pathogen (53 – 153)	Episodes
India 1989 [18]	New Delhi	1984 1 yr	< 2 yrs, no blood in stool, weight loss	Hospital admissions	Consecutive cases	92	Children
India 1992 [19]	New Delhi	1988 28 mths	< 3 yrs, no blood in stool, weight for height = 90% of standard	Hospital admissions	Not described	81	Children
India 1999 [22]	Slum New Delhi	Unclear Unclear	< 3 yrs	Active household surveillance	All cases identified	115	Episodes
Brazil 1990 [26]	Urban slum, Fortaleza	1984 28 mths	< 5 yrs	Active household surveillance	All cases identified	40	Episodes
Brazil 1995 [29]	Fortaleza	1988 32 mths	< 3 yrs, no antibiotics in past 72 hrs	Hospital admissions	Not described	56	Children
Brazil 2000 [4]	Shanty town Fortaleza	1989 45 mths	< 4 yrs	Active household surveillance	All cases identified	88	Episodes
Bangladesh 1988 [14]	Dhaka	1983 3 yrs	< 5 yrs	Outpatient attendances	Systematic every 25^th ^case	410	Children
Bangladesh 1991 [15]	Dhaka	1983 3 yrs	1 mth to 3 yrs	Outpatient attendances	Systematic every 25^th ^case	Varied by pathogen (391–445)	Children
Bangladesh 1998 [31]	Dhaka	Unclear 3 yrs	3 mths to 2 yrs, no blood in stools, no antibiotics, anti-diarrhoeals or systemic infection	Outpatient attendances	Not described	138	Children
India 1995a [20]	Madras	Unclear Unclear	< 5 yrs	Hospital admissions	Not described	100	Children
India 1995b [21]	Amritsar	Unclear Unclear	< 3 yrs	Hospital admissions	Not described	150	Children
India 2001 [23]	Varanasi	1998 15 mths	1 to 5 yrs, weight loss or failure to gain weight	Hospital admissions & outpatient attendances	Randomly	57	Children
Guatemala 1992 [27]	Rural indigenous community	1988 20 mths	< 3 yrs, watery diarrhoea for 14 to 18 days	Active household surveillance	All cases identified	49	Children
Mexico 2003 [28]	Mexico City	1997 Unclear (24–36 mths)	mean age 16.6 mths, no specific diagnosis	Hospital admissions	Not described	89	Children
Cambodia 1992 [24]	Resettlement camp, Thailand Cambodia border	1989 6 mths	< 5 yrs, with temperature over 38°C, colic or vomiting	Outpatient attendances	Consecutive cases	79	Children
Vietnam 1992 [25]	Hanoi	1988 1 yr	< 3 yrs	Hospital admissions	Not described	83	Children
Zambia 1995 [32]	Lusaka	Unclear 9 mths	15 mths to 5 yrs; diarrhoea for at least 30 days	Hospital admissions	Consecutive cases every alternate day	90	Children
Zambia 2001 [30]	Lusaka	1998 26 mths	Inpatient in nutrition ward, median 18 mths, no blood in stool	Hospital admissions	Consecutive cases	194	Children

**Table 5:** Characteristics of included studies: comparison groups without diarrhoea

Study report	Location	Source of cases	Number of cases	Source of comparison group without diarrhoea	Matching of comparison group	Number of children without diarrhoea
Bangladesh 1992a [16]	Matlab (rural)	Active household surveillance	Varied by pathogen (68 – 184)	Same community study cohort	Age and proximity of residence	Varied by pathogen (67 – 164)
Bangladesh 1992b [17]	Mirzapur (rural)	Active household surveillance	Varied by pathogen (53 – 153)	Same community study cohort	Age and proximity of residence	Varied by pathogen (46 – 213)
India 1989 [18]	New Delhi	Hospital admissions	92	Other wards with non-gastro-intestinal illnesses	Age and nutritional status	92
India 1992 [19]	New Delhi	Hospital admissions	81	Nutrition clinic with failure to thrive and weight for height = 90% of standard	Not matched	32
India 1999 [22]	Slum of New Delhi	Active household surveillance	115	Same community study cohort	Age, nutritional status and proximity of residence	115
Brazil 1990 [26]	Urban slum, Fortaleza	Active household surveillance	40	Same community study cohort	Not matched	38
Brazil 1995 [29]	Fortaleza	Hospital admissions	56	Children on other wards	By age	42
Brazil 2000 [4]	Shanty town, Fortaleza	Active household surveillance	88	Same community study cohort	By age and sex	443

### Locations and settings

Ten studies were conducted in the South-East Asia region; four in Bangladesh[15-18,32] and six in India [19-24]. Five were conducted in the Americas, including three in Brazil[4,27,30], one in Guatemala[28] and one in Mexico[29]. Two were conducted in the Western Pacific region, one on the border between Thailand and Cambodia[25], and one in Vietnam[26]. Two studies were conducted in the African region, both in Zambia[31,33].

Some studies were conducted within particularly vulnerable communities, including three in areas described as urban slums or shantytowns[4,23,27], one in a rural indigenous community[28] and one in a resettlement camp for refugees[25].

### Participants

The included studies all involved between 40 and 410 children with persistent diarrhoea; 11 studies had less than 100 participants[4,19,20,24-30,33], seven had more than 100 but less than 200 participants [17,18,21-23,31,32], and one had more than 200 participants [15,16].

Fourteen studies included children from birth to a specific age; in one study it was two years[19], in six it was three years[20,22,23,28,30], in one it was four years[4], in five it was five years[15-17,21,25,27] and in one it was six years[18]. Others included children aged one to 36 months[16], three to 24 months[32] one to five years[24], and 15 months to five years[33]. Two did not state an age range for inclusion, but presented the mean and median participant ages, respectively 16.6 months[29], and 18 months[31]. There were no apparent patterns of age range inclusion by region.

Five studies[19,20,28,31,32] (three in Southeast Asia, one in the Americas, one in the African region) excluded children with bloody diarrhoea, or included only those with watery diarrhoea. Three included children with all types of persistent diarrhoea, while the other studies did not describe the type of diarrhoea included. One study in the African region included only children with diarrhoea over 30 days in duration[33], one in the Americas excluded those with diarrhoea that had persisted for longer than 18 days[28].

Five studies included only children with additional signs and symptoms; two in Southeast Asia stated that the children must have lost weight or failed to gain weight during the episode[19,24], two (one in Southeast Asia, one in Africa) that the children should have specific signs of under nutrition [20,31], one in the Western Pacific specified that children should also have either a rectal temperature of over 38°C, colic or vomiting[25]. One study in the Americas excluded children with specific diagnoses such as caeliacs disease or short bowel syndrome[29], one in South East Asia excluded children with systemic infections[32] and another in Africa excluded children with neurological diseases[31]. Two studies excluded children who had recently received antibiotics [30,32].

Two studies; both from Africa, looked specifically at children who were HIV positive, and presented data on HIV positive and HIV negative individuals separately. The data is not presented separately in this review, as in one study only 24 individuals were HIV positive[33] and the other reported no significant differences in pathogen frequency between the HV positive and HIV negative groups[31].

### Sources of participants

Six of the included studies employed active surveillance to identify all cases of persistent diarrhoea within a specific cohort of children in a defined community, including three from South-East Asia[17,18,23] and three from the Americas[4,27,28]. Three sampled children attending hospital outpatient departments, including children who were also subsequently admitted to the wards, two in South-East Asia[15,16,32] and one in the Western Pacific[25]. Nine sampled only children who were already admitted to a hospital ward, four in South-East Asia [19-22], two in the Americas[29,30], two in Africa[31,33] and one in the Western Pacific[26]. One study in South-East Asia included both inpatients and outpatients treated at a hospital[24].

### Sampling strategies

All six of the community cohort studies included all the children identified with persistent diarrhoea over the duration of the project. Four of the studies recruiting patients at healthcare facilities also sampled all consecutive eligible patients[19,25,31,33]. One study included a random sample (method of randomisation not stated)[24] and one used a systematic sampling method (every 25^th ^patient)[15,16]. Reports of the remaining studies did not describe their sampling methods.

### Date of studies

Most of the included studies were conducted over ten years ago, and none presented data collected after 2000. Taking the mid-year of the data collection period as the reference point, three collected data in 1985 or before [15,16,19,27], eight collected data between 1986 and 1990 [4,17,18,20,25,26,28,30], and three collected data between 1996 and 1999 [24,29,31]. Five study reports [21-23,32,33] did not state the date of the data collection, three were published 1995, one was published 1998, and one was published in 1999. There did not appear to be any pattern of date of study by WHO region.

### Duration of studies

Most of the included studies collected data for a period of one year or more. One collected data for six months[25], and one over a period of nine months[33]. Six collected data for periods of whole years: three for one year [17,19,26], one for two years [18] and two for three years[15,16,32]. Six collected data for other periods of time, between one and four years. Three study reports did not give any information about dates or durations of data collection [21-23]. There did not appear to be any pattern of duration of data collection period by WHO region.

### Units of analyses

Five of the community cohort type studies used episodes as the unit of analysis, with children in both the persistent diarrhoea and comparison groups (where used) often included in the analysis more than once. One of the studies recruiting children from a community surveillance project was reporting baseline data for a clinical trial, and therefore included individual children only once[28]. The remaining studies presented data as for individual children, although none reported procedures to exclude children who presented to the facility more than once during the study period.

### Comparison groups without diarrhoea

Eight study reports presented pathogen frequency data on an appropriate comparison group of children without diarrhoea, five in south-East Asia[17-20,23] and three in the Americas[4,27,30]. One study described a comparison group but did not present any relevant data[21]. In six studies, the comparison participants were matched to participants with persistent diarrhoea using specific criteria[4,17-19,23,30], in two the diarrhoea and comparison groups were not matched[20,27]. Five of the studies with comparison groups were community cohort studies that recruited the comparison group from the same community[4,17,18,23,27]. The other three studies were based at health facilities and used a comparison group of children attending or admitted to the hospital with illnesses other than diarrhoea.

### Quality of reporting for laboratory procedures

Table 6 gives a summary of the assessed quality of reporting of the laboratory procedures used in the study, plus an indication of whether there was any mention of antimicrobial susceptibility testing of bacteria being carried out, and whether quality control procedures were described. The quality of reporting varied widely across studies in all WHO regions, with one study describing none of the procedures[24], while two reported six out of seven[18,25]. Two trials tested only for parasites and used three out of the three possible procedures on our checklist[23,33]. Only two studies reported testing bacterial isolates for antimicrobial susceptibility[18,24,25], and two described quality control procedures[23,33].

**Table 6:** Laboratory Procedures described or referenced in the included study reports

	Samples	Parasite detection	Bacterial culture	Bacterial identification	Virus detection	Quality control	Antimicrobial susceptibility testing
Bangladesh 1988 [14]	X	X	X	X	√	X	X
Bangladesh 1991 [15]	√	-	√	√	√	X	X
Bangladesh 1992a [16]	√	√	√	√	-	X	X
Bangladesh 1992b [17]	√	√	√	√	√	X	√
Bangladesh 1998 [31]	√	√	√	√	√	X	X
India 1989 [18]	√	√	√	√	√	X	X
India 1992 [19]	√	X	√	√	X	X	X
India 1995a [20]	X	X	√	√	-	X	X
India 1995b [21]	√	√	√	√	-	X	X
India 1999 [22]	√	√	-	-	-	√	-
India 2001 [23]	X	X	X	X	-	X	X
Cambodia 1992 [24]	√	√	√	√	√	X	√
Vietnam 1992 [25]	X	X	X	X	√	X	X
Brazil 1990 [26]	√	√	√	√	√	X	X
Brazil 1995 [27]	√	√	√	√	√	X	X
Brazil 2000 [4]	√	√	√	X	√	X	X
Guatemala 1992 [29]	√	√	√	√	√	X	X
Mexico 2003 [28]	X	√	√	√	-	X	X
Zambia 1995 [32]	√	√	-	-	-	√	-
Zambia 2001 [30]	X	√	X	X	-	X	X

### Potential pathogen prevalence

Table S1 in Additional file 1 (link) shows, for each study, the percentage of cases testing positive for different pathogens in the stool, and associated 95% confidence limits, in children with persistent diarrhoea, and where available, children without diarrhoea. The estimated prevalence of each pathogen tended to be very imprecise, due to small sample sizes. For studies with comparison groups without diarrhoea, there was no significant difference in prevalence of any pathogen between the two groups.

Under Table S1 in Additional file 1 we list the *E. coli *types tested for in each study, using the category conventions stated by authors. All of the studies included ETEC, most also included EPEC; others included AggEC or EIEC; two studies included EHEC. In two studies[17,20], it was uncertain whether one category of *E. coli *referred to EPEC or EHEC; in these cases it was assumed to be EPEC. No percentages were calculated for individual *E. coli *types as different naming conventions would have made comparison between studies impossible. Two different sets of *E. coli *estimates are presented: those based on the upper estimate of the range, and those based on the lower estimates. Only two studies tested for isospora[31,33] and two tested for enteric adenovirus[4,19]. One study tested for *Norovirus *[4] but was excluded from the analysis because the sample size was less than 30. No studies tested for *Astrovirus *or *Cyclospora*. Two studies tested specifically for EHEC and found no EHEC infections[4,19].

As the prevalence of individual organisms did not show extreme variations between studies, we combined the data across studies to provide an approximate indicator of prevalence across studies.

Study findings combined by WHO region are shown in Table S2 in Additional file 1. Table S2 in Additional file 1 shows the number of studies testing for each pathogen, the combined number of persistent diarrhoea cases tested, the weighted mean percentage across studies of cases testing positive, and the range between studies. There were no apparent differences by region, although there were some differences between studies, for example, rotavirus was detected in between 0% and 20% of children.

**Figure 1:**
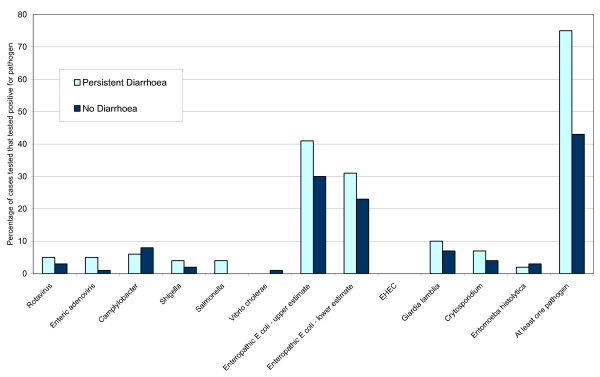
**Weighted mean percentage across all studies of cases testing positive for each pathogen included in the review**.

Figure 1 illustrates in chart form the weighted mean percentage across all studies of cases testing positive for each pathogen included in the review. It is apparent that the stool samples of children with persistent diarrhoea contained a range of pathogens. With the exception of enteropathic *E. coli *types, each pathogen was found in less than 10% of children across studies; in the case of *E. coli *the estimated range was between 25% and 33%. In studies that tested for a reasonably comprehensive range of pathogens, 65% of cases had some kind of pathogen detected in the stool, varying widely between studies from 36% to 88%.

For each pathogen in each region, a median percentage of cases testing positive across the studies was also calculated. These are not presented, as in most cases they were less than 5% (absolute difference) either side of the mean. The only exception was the lower estimate for *E. coli *in the Americas, where the mean was 35% and the median was 25%.

### Comparative prevalence of potential pathogens

For studies with an appropriate comparison group, Table 7 shows the number of studies testing for each pathogen, total sample size, and weighted mean percentage across studies of cases and comparisons testing positive for each pathogen, separated by WHO region. There were no studies with a comparison group from the Western Pacific region.

**Table 7:** Summary of studies with control group: number of participants testing positive for different pathogens by region

	South-East Asia					The Americas					All				
	Studies	Persistent diarrhoea		No diarrhoea		Studies	Persistent diarrhoea		No diarrhoea		Studies	Persistent diarrhoea		No Diarrhoea	
		N	Weighted Mean	N	Weighted Mean		N	Weighted Mean	N	Weighted Mean		N	Weighted Mean	N	Weighted Mean
Viruses															
Rotavirus	4	496	2%	501	1%	3	162	12%	162	7%	7	658	5%	663	3%
Enteric adenovirus	1	92	1%	92	2%	1	66	11%	82	0%	2	158	5%	174	1%
															
Bacteria															
Campylobacter	4	489	8%	375	9%	2	96	0%	80	0%	6	585	6%	455	8%
Shigella	4	496	5%	501	3%	3	184	3%	520	2%	7	680	4%	1021	2%
Salmonella	3	326	6%	337	0%	3	184	0%	520	0%	6	510	4%	857	0%
Vibrio cholerae	2	317	1%	373	1%	1	88	0%	440	0%	3	405	0%	813	1%
*All Enteropathic															
E coli – upper estimate	4	386	32%	329	27%	3	164	63%	75	38%	7	550	41%	604	30%
*All Enteropathic															
E coli – lower estimate	4	386	26%	328	22%	3	164	41%	275	25%	7	550	31%	603	23%
EHEC	1	92	0%	92	0%	1	51	0%	51	0%	2	143	0%	143	0%
															
Parasites															
Giardia lamblia	5	504	8%	540	7%	3	184	14%	522	7%	8	688	10%	1062	7%
Cryptosporidium	2	232	1%	280	4%	3	167	16%	369	5%	0	399	7%	649	4%
Entamoeba histolytica	5	504	2%	541	4%	3	184	1%	522	1%	5	688	2%	1063	3%
															
At least one pathogen	1	92	73%	92	22%	2	112	76%	233	60%	3	204	75%	325	43%

**Figure 2:**
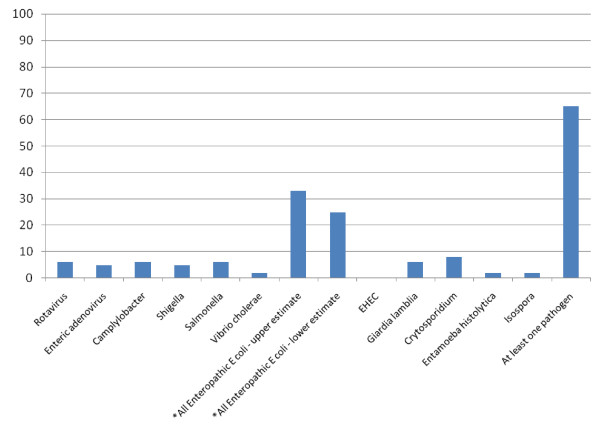
**Weighted mean percentage of cases tested that tested positive for different pathogens in children with persistent diarrhoea and comparisons without diarrhoea**.

Figure 2 illustrates in chart form the weighted mean percentages of cases tested that tested positive for different pathogens within the persistent diarrhoea groups and comparison groups without diarrhoea in all eight studies with a comparison group. As described in the methods section, the data from the comparison group in each study was weighted according to the sample size of the group with persistent diarrhoea.

It is apparent from Table 7 and Figure 2 that the frequency of detection of any kind of enteric pathogen was higher in children with persistent diarrhoea, but for individual pathogens any differences were very small (although differences between groups were larger in the Americas than in other locations).

## Discussion

We were able to identify, retrieve and assess papers describing 19 studies, of which eight also presented data for an appropriate comparison group without diarrhoea. However, other relevant studies may have been published, but missed by the search. Some of the included studies had main purposes that were different from the purpose of this review, and titles that reflected this, for example 'Endoscopic and histopathological evaluation of pre-school children with chronic diarrhoea' and 'Oral gentamicin is not effective treatment for persistent diarrhoea'. We identified two reports only through the search process for another review, and three through an informal search of 'Google Scholar'. We can therefore suppose that not all published studies presenting relevant data are indexed in such a way that even quite wide systematic electronic searches, such as the one we performed, can easily identify. In addition, we know of four studies that may possibly be relevant but that we were unable to obtain within the UK.

The included studies were conducted in a range of geographic locations, including South-East Asia, the Americas, Western Pacific and African Regions, some in contexts where children may have a particularly high risk for persistent diarrhoea, including slum areas and a resettlement camp for refugees. The two studies carried out in Africa were both based within the same hospital in Zambia; these were the only studies to specifically include children who were HIV positive; the sample sizes were too small to assess whether the patterns of enteric infection were different in HIV positive and HIV negative children.

The included studies used a range of methods and settings, including surveys of children attending clinics or admitted to hospital, baseline data collection on children recruited for a clinical trial and community surveillance projects seeking to actively identify children with persistent diarrhoea. The units of analyses also differed; some studies included individual children only once, while some included all separate cases, whether or not they were identified in the same child.

This review can only give an approximate indication of the actual frequency of different pathogens, for several reasons. Most studies had small sample sizes, so estimates from individual studies are imprecise, and different studies tested for different pathogens, therefore even when study results were combined, the sample size for some organisms was still small. Some of the pathogens that we considered to be important were not tested for in any of the studies. In the case of *E. coli*, evolving knowledge meant that different classification systems were used in different studies, making it impossible to combine data on type of *E. coli *across studies. Some studies included more types of *E. coli *than others, automatically leading to higher estimated prevalences for all enteropathic *E. coli *types combined. The reporting of microbiological procedures was generally poor, so it is unknown whether optimum testing methods were used. The majority of studies were more than ten years old; the relative prevalence of different pathogens in persistent diarrhoea may have changed since that time, and tests for different pathogens may have become more accurate. In addition, the duration of many studies was not a multiple of 12 months, so the results could have been affected by seasonal variations. In regards to the comparison groups without diarrhoea, in some studies the children may not have been typical of children without diarrhoea in the community, as they were hospital inpatients.

We used simple weighted means to combine data; this could have given undue weight to larger studies which may not have had findings typical of the other studies. However, we also calculated medians and found them very similar to the means, suggesting that any such effect, if present, was not large. We also combined data extracted from studies using different methodologies, including studies presenting data based on individuals and those presenting data on episodes, where some individuals were included more than once. This was a pragmatic decision based on the fact that in some studies it was unknown whether each case presented related to a different individual or not. The methodological difficulties may have been important if this review were to claim to represent a 'typical' population of young children with persistent diarrhoea in a low or middle income country; however the wide range of study dates, settings and inclusion criteria does not allow this. Frequencies of different pathogens could feasibly vary according to study setting, particularly in community setting compared with a hospital setting, where the severity of disease is likely to differ. We did not consider it worthwhile to undertake separate analyses for studies carried out in different settings, because the wide range of other differences between studies meant that the results of any such analyses would have been very difficult to interpret.

Despite the limitations of the included studies, and the methods used to combine their results, the findings are useful, as they highlight the range of pathogens that may be associated with persistent diarrhoea, the potential of these pathogens to vary in frequency by time and place, and the uncertainty within populations and individuals that any particular organisms are causal in persistent diarrhoea.

In individual studies comparing children with persistent diarrhoea and without diarrhoea, there were no significant differences between the groups in the prevalence of any individual enteric pathogen, and any differences were small even when all the studies' results were combined. A higher proportion of children with persistent diarrhoea tested positive for any pathogen, but the frequency of detection was also high in children without diarrhoea; across studies that tested for a comprehensive range of organisms, 65% of children with persistent diarrhoea tested positive for at least one pathogen, compared with 43% of children without diarrhoea. In every study, a proportion of children with persistent diarrhoea did not test positive for any enteric pathogen, although no study included tests for all relevant micro-organisms.

Children with persistent diarrhoea and without diarrhoea carried a wide range of enteric pathogens, including viruses, bacteria and parasites, and the rates of each varied considerably between studies, although there were no apparent differences between WHO regions. The frequency of most pathogens was low, tending to vary in children with persistent diarrhoea from 0% or 1% to between 3% and 20% between studies. *E. coli *was an exception, being much more common, detected in between 25% to 33% of cases across studies, 6% to 91% between studies. This does not take into account the different classes of *E. coli *tested for in different studies.

## Conclusion

There is no evidence that any particular pathogen or type of pathogen is associated with persistent diarrhoea in children under the age of six in low and middle income countries. There is therefore no evidence to justify routine antimicrobial use for children with persistent diarrhoea of unknown cause, in keeping with current guidelines. Even in cases where diagnostic facilities are routinely available, pathogens detected in children with persistent diarrhoea may not be the cause of the illness.

The data currently available on the frequency of different enteric pathogens found in the stools of children with persistent diarrhoea and without diarrhoea is limited in its applicability and generalisability, especially in the range of pathogens being tested for and the locations of the studies. Better quality data, gathered from a range of settings in low and middle income countries throughout the world (including locations where routine microbiological testing is not usually available), and repeated at set time points, could inform health staff in the region of the types of pathogens they should be alert to, and also inform future policy and practice, both locally and globally.

Further good quality studies are needed. The analysis of already available data, for example from hospitals or clinics where routine microbiological testing is used, might also be useful. Studies should ideally run for at least one year, and present data collected over periods of exactly 12 months, or multiples of 12 months, particularly in areas with seasonal climates. Sample sizes should be large enough to give reasonably precise estimates of pathogen frequency, given the wide range of pathogens that may be found, and the low frequency of some of them. Authors should report the study design and microbiological methods in sufficient detail to enable their quality to be assessed. The types of pathogens tested for in these studies should be standardised as far as possible, while remaining flexible to allow the inclusion of new or previously rare or unsuspected pathogens. The classification of enteropathic *E. coli *should also be standardised if possible, concentrating on clinically useful distinctions between the types.

## Abbreviations

ETEC: Enterotoxigenic E. coli; EIEC: Enteroinvasive E. coli; EPEC: Enteropathogenic E. coli; EaggEC: Enteroaggregative E. coli; EHEC: Enteroheamorrhagic E. coli.

## Competing interests

The authors declare that they have no competing interests.

## Authors' contributions

PG conceived of the study, secured its funding, participated in the design, assisted with the interpretation of data and helped to draft the manuscript. KA wrote the review protocol, inspected the initial search results for potentially relevant publications, selected studies for inclusion, extracted data, undertook the data analysis and drafted the manuscript. RS inspected the initial search results for potentially relevant publications, extracted data, and assisted in the interpretation of data. CAH participated in the design, particularly advising on which pathogens were important and how to handle data on *E. coli*, and assisted in the interpretation of the findings. PG, KA and RS all contributed to and approved the final manuscript. CAH died before the completion of the manuscript. He was a Professor within the Department of Infections and Host Defence, School of Medicine, University of Liverpool. All three living authors read and approved the final manuscript.

## Pre-publication history

The pre-publication history for this paper can be accessed here:

http://www.biomedcentral.com/1471-2334/9/88/prepub

## Supplementary Material

Additional file 1 Supplemental tables. Click here for file
